# Assessing the Feasibility and Acceptability of Smart Speakers in Behavioral Intervention Research With Older Adults: Mixed Methods Study

**DOI:** 10.2196/54800

**Published:** 2024-08-30

**Authors:** Kelly Quinn, Sarah Leiser Ransom, Carrie O'Connell, Naoko Muramatsu, David X Marquez, Jessie Chin

**Affiliations:** 1 Department of Communication University of Illinois Chicago Chicago, IL United States; 2 Division of Community Health Sciences School of Public Health University of Illinois Chicago Chicago, IL United States; 3 Department of Kinesiology and Nutrition University of Illinois Chicago Chicago, IL United States; 4 School of Information Sciences University of Illinois Urbana-Champaign Urbana, IL United States

**Keywords:** smart speakers, physical activity, older adults, behavioral health, intervention, smart device, smart devices, conversational agent, physical activities, behavioral intervention, intervention research

## Abstract

**Background:**

Smart speakers, such as Amazon’s Echo and Google’s Nest Home, combine natural language processing with a conversational interface to carry out everyday tasks, like playing music and finding information. Easy to use, they are embraced by older adults, including those with limited physical function, vision, or computer literacy. While smart speakers are increasingly used for research purposes (eg, implementing interventions and automatically recording selected research data), information on the advantages and disadvantages of using these devices for studies related to health promotion programs is limited.

**Objective:**

This study evaluates the feasibility and acceptability of using smart speakers to deliver a physical activity (PA) program designed to help older adults enhance their physical well-being.

**Methods:**

Community-dwelling older adults (n=18) were asked to use a custom smart speaker app to participate in an evidence-based, low-impact PA program for 10 weeks. Collected data, including measures of technology acceptance, interviews, field notes, and device logs, were analyzed using a concurrent mixed analysis approach. Technology acceptance measures were evaluated using time series ANOVAs to examine acceptability, appropriateness, feasibility, and intention to adopt smart speaker technology. Device logs provided evidence of interaction with and adoption of the device and the intervention. Interviews and field notes were thematically coded to triangulate the quantitative measures and further expand on factors relating to intervention fidelity.

**Results:**

Smart speakers were found to be acceptable for administering a PA program, as participants reported that the devices were highly usable (mean 5.02, SE 0.38) and had strong intentions to continue their use (mean 5.90, SE 0.39). Factors such as the voice-user interface and engagement with the device on everyday tasks were identified as meaningful to acceptability. The feasibility of the devices for research activity, however, was mixed. Despite the participants rating the smart speakers as easy to use (mean 5.55, SE 1.16), functional and technical factors, such as Wi-Fi connectivity and appropriate command phrasing, required the provision of additional support resources to participants and potentially impaired intervention fidelity.

**Conclusions:**

Smart speakers present an acceptable and appropriate behavioral intervention technology for PA programs directed at older adults but entail additional requirements for resource planning, technical support, and troubleshooting to ensure their feasibility for the research context and for fidelity of the intervention.

## Introduction

### Background

The use of behavioral intervention technologies (BITs) in research has proven to be feasible and efficacious in a wide variety of settings [1], extending the range of research into new geographies and populations that were previously difficult to reach and by providing new media with which to develop and deliver interventions and record data [2]. Advances in artificial intelligence and computational linguistics have created a new class of technologies that can be used for these purposes. Powered by artificial intelligence and made accessible through voice user interfaces (VUIs), smart speakers or voice-activated personal assistants, such as Amazon’s Alexa and Google’s Nest Home, are widely available and readily acceptable to older adults [3]. Because of their utility and features, smart speaker technologies have become a focal point in gerontological and health research [4,5]. While attention has been placed on the use of websites, software, mobile apps, and sensors as intervention delivery mechanisms [6], less attention has been placed on the use of smart speakers as a BIT, especially for older adult populations [5].

Hermes et al [7] argue that BITs hold unique characteristics that should be evaluated distinctly as part of traditional implementation outcomes, with an emphasis placed on the evaluation of BIT at the consumer or participant level for factors such as acceptability and adoption. We further argue that BITs often comprise both a delivery technology, such as a smart speaker, and an underlying software application, which is the intervention itself. By conducting independent evaluations of intervention hardware and delivery technology and intervention software applications, a more accurate evaluation of implementation outcomes can be made.

This article aims to report on the acceptability and feasibility of using smart speakers to deliver an in-home physical activity (PA) intervention among a sample of older adults aged ≥65 years. Using data collected from surveys and interviews, along with researcher field notes and device logs, we focus on the evaluation of the smart speaker device, and not the intervention application, as a BIT delivery mechanism in a 10-week pilot study that used Google Nest Home Mini smart speakers. The contribution of this study is 2-fold. First, it examines the feasibility and acceptability of smart speakers as an emerging component of BIT delivery systems, independently of an intervention assessment. Second, it examines the appropriateness of smart speaker technology for use in PA interventions for older adults.

### Smart Speaker Basics

Smart speakers use a VUI to aid users in navigating everyday tasks, such as finding information, scheduling events, setting timers and alarms, and playing media [8,9]. The VUI language processing system, also called a voice assistant or conversational agent, is the defining characteristic of the smart speaker. To accomplish tasks by voice, smart speakers integrate several different technologies into a single device to leverage dialogue capabilities: these include subsystems for voice recognition, natural language processing (understanding and generation), and cloud-based data processing. Typically activated using a wake word or phrase, such as “Hey Google” or “Alexa,” smart speakers remain in a state of ambient listening or are always “on,” waiting for the users to initiate a conversation or command. Energy-efficient processors passively process, or “listen,” for the wake word, buffering and rerecording within the device without transmitting or storing any information [10].

**Figure 1:**
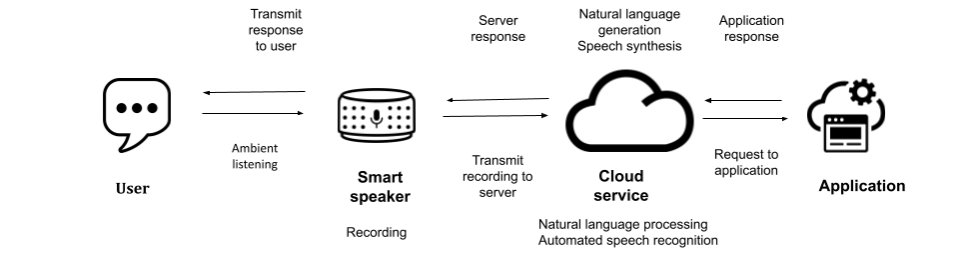
Illustration of smart speaker operation.

As illustrated in Figure 1, once a wake word is detected, the device is triggered to begin actively recording [11], transmitting recorded requests to the device maker’s cloud-based service to decipher users’ speech [12]. Cloud-based data processing and storage alleviates the need for the device to be capable of speech recognition [11] or file storage [13], and data are transmitted seamlessly between the device and the cloud. The audio transmission is deciphered into commands using a natural language processing algorithm, and an appropriate response is generated using speech synthesis, then sent back to the smart speaker to be conveyed to the user [12,14].

To enhance device utility, most platforms like Google and Amazon encourage personalization of the activities that can be performed on their devices. Users are urged to create user and voice profiles and to share personal information like home and work addresses, credit card numbers, calendars, account logins, transportation modes, and nicknames. This information is then used to streamline activities that can be facilitated through the device, such as purchasing items, setting calendar reminders, and generating shopping lists. In another form of customization, Google and Amazon provide developers the ability to build and market add-on applications called skills (for Amazon’s Alexa) and actions (for Google Assistant), which augment available native applications. These custom actions can be designed to support research activities by recording data, supporting intervention activities, or reminding participants to pursue specific actions.

### Older Adults and Technology Acceptance

Technology has become increasingly important to everyday life, yet older adults can often trail behind in the adoption of new technology because of limited experience and a lack of necessary skills [15,16]. While age-related gaps in internet use narrowed significantly since the pandemic, older adults still lag behind other age groups in both use and access to broadband connections, as compared with those in younger age groups [17,18]. In part, this may be due to experience with technology in the workplace [19], but declines in physical and cognitive abilities and limitations in the performance of instrumental activities of daily living may also lead to decreased use [20,21]. Although the use of technology by older persons can often enhance perceptions of life quality [22,23], socioeconomic factors, such as lower income and education levels, compound age-related differences [24].

VUIs represent a class of technologies that are readily accepted by older adults and perceived as easier to learn and use than keyboard interfaces [3,25,26]. Because VUIs do not rely on vision or touch, they are accessible to those with visual or fine motor degradation, mobility impairment, and disability [27]. In addition, older adult users often build companionship with VUIs, which results in positive experiences that may not only lead to reduced loneliness and increased independence [28] but also may help to overcome frustration with technological errors [29]. Processes of technology acceptance by older adults often reflect the dynamics of technology adoption and use described by the Technology Acceptance Model and its derivatives [30], including the widely accepted Unified Theory of the Acceptance and Use of Technology (UTAUT) model [31].

### Older Adults and PA

The health benefits of PA for older adults are well documented [32], and its importance in supporting healthy aging cannot be overstated. PA slows age-related declines in functional abilities and helps to maintain physical and mental capacities in such diverse areas as muscle strength, cognitive functioning, disease prevention, and anxiety and depression reduction [33]. Despite these benefits, older adults report high levels of sedentary behavior [34]. Environmental contexts, such as weather, accessibility because of distance, and cost and affordability, are cited as factors by older adults for not being physically active [35]. It is often challenging for older adults to adopt and adhere to a PA regimen [36,37], and mobility challenges sometimes limit the ability to regularly participate in community-based programs because of accessibility issues [38].

Behavioral interventions to increase PA among older persons are largely successful in increasing levels of PA, and studies have shown that older individuals are more likely to continue PA programs in home-based settings [39,40]. Internet-based PA programs are both cost-efficient [41] and effective in producing behavioral change [42], and home-based programs have better adherence rates than community-based programs [40]. Technology-based interventions have been effective in producing a change in PA behaviors when compared with traditional mechanisms, such as usual care, minimal contact, waitlist control groups, in-person, or other nontechnology interventions [43]. Taken together, these factors suggest that a home-based PA program facilitated by technology, such as a smart speaker, could support the PA readiness of sedentary older adults. This study contributes to the extant literature by reporting on the acceptability and feasibility of smart speakers to deliver PA programming among a sedentary group of older adults.

### Smart Speakers as a BIT

Smart speakers are emerging as a locus in behavioral intervention delivery systems [5]. Smart speakers can be deployed in participants’ local environments and enable interventions to be delivered remotely, thereby reducing barriers to administration and adherence. When evaluating the use of technology in implementation research, Hermes et al [7] argue that criteria used to evaluate intervention implementations [44] should be applied to BITs and the strategies used to guide their use apart from processes used to evaluate intervention implementation, as by conducted these separately a more accurate evaluation of implementation outcomes can be made.

It is relevant to note that the Hermes et al [7] approach ignores potential distinctions between an intervention mechanism, which may be a technology, such as a software program or application through which the intervention is delivered, and its underlying hardware and delivery infrastructure, which may include a device, such as an internet-enabled watch or smart speaker through which the intervention software operates, as well as the Wi-Fi or internet signal on which it is dependent. We argue that distinctions between each of these elements are important to make when evaluating the feasibility and acceptability of a BIT as well, as challenges and successes may occur with any of the components. Moreover, it is relevant to consider a participant’s ability to distinguish between these elements when undertaking this analysis to ensure appropriate identification of evaluation criteria. For example, studies on smart speakers used for general purposes have suggested that some users fail to make distinctions between the various components of service delivery, such as the voice interface and the device [45,46], which leads to conflation between perceptions of each element.

Five criteria in particular are recommended for evaluation from the perspectives of the participant or consumer and providers or researchers [7,44]:

1. Acceptability, or the extent to which a technology is useful or satisfactory.
2. Adoption, or the intention to use the technology.
3. Appropriateness, or perception that the technology fits, is relevant or compatible with the context of its use.
4. Feasibility, or the extent to which a technology can be successfully used in a specific context.
5. Fidelity, or evidence that technology can be delivered as intended.

When evaluating a BIT at the level of the consumer or research participant, the outcomes of acceptability, feasibility, and adoption are most commonly measured through models of technology adoption [7]. The UTAUT model [31] is one of the most widely used theories of technology adoption [47], connecting the concepts of acceptability and feasibility to a third important criterion from the user’s perspective: adoption. UTAUT argues that a causal relationship exists between users’ perceptions of technology and their intention to use it. It specifically identifies 4 constructs—expectations of a technology’s performance, the ease with which it can be used, social influence, and facilitating conditions—and links these to a user’s intention to use a particular technology, which, in turn, is strongly correlated with its use. UTAUT subscales regarding performance and effort expectancy have been proven reliable with respect to the use of a wide variety of technologies in older adult populations, including email and social media [48], tablet computers [49], and remote health care or telehealth applications [50].

Studies on older adults’ use of technology have found that expectations of a technology’s performance and perceptions of the amount of effort that will be required to use technology are powerful incentives for new technology adoption [51,52]. Although the feasibility of using smart speakers among older adult populations to improve well-being has been examined in several recent studies [25,28,53], their use to explore specific interventions is more limited, especially among older adult populations.

## Methods

### Study Design

Participants were engaged in a 10-week, evidence-based, internet-based PA program that used artificial intelligence to guide activities from October 2020 through January 2022. Of note, the time frame of the study coincided with the COVID-19 pandemic and consequent lockdowns. Because all in-person leisure activities had ceased, the use of alternative delivery mechanisms for exercise programming, such as smart speakers, was potentially attractive. Though the study had been initially designed and planned to include an in-person orientation to the smart speaker and PA application, the pandemic necessitated that all participant interactions be carried out in a virtual context because of the particularly vulnerable nature of the target population. Consequently, all participant interactions, including onboarding and offboarding, weekly check-ins, and interviews, were conducted remotely via Zoom when possible and alternatively via phone calls.

A PA application was developed by the research team to run on Google Nest Home Mini speakers, which replicated components of the Healthy Moves for Aging Well program [54]. A description of the activities included in the PA application can be found in Multimedia Appendix 1. Initial feasibility and acceptance of the PA application were validated in a pilot user study before field deployment [39].

To ensure that participants had internet access, we distributed Wi-Fi hot spots together with the smart speakers. The hot spots also enabled the research team to perform the initial speaker setup and installation of the PA application before the first session. This simplified the orientation process for participants, as the devices merely needed to be connected to power for participant use.

**Figure 2:**
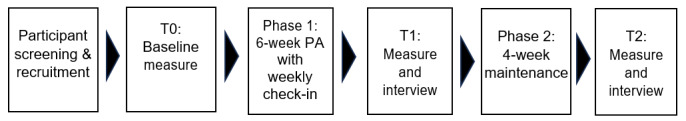
Study design. PA: physical activity; T0: baseline; T1: 6 weeks and end of phase 1; T2: 10 weeks and end of phase 2.

Recruitment was aided by a partner senior-living organization located in a large suburban Midwestern county, which disseminated recruitment materials to both their independent living facility residents and via community programming information channels. The research team consisted of 4 individuals (1 principal investigator and 3 graduate research assistants, all adult women), certified by the Collaborative Institutional Training Initiative Program [55]. The study design is illustrated in Figure 2.

After enrollment, participants were sent a parcel containing written instructions regarding the use of the smart speaker, PA program application, and Wi-Fi hotspot; a smart speaker; a Wi-Fi hot spot for in-home internet connection diary materials; and consent documents. Initial participant meetings were conducted by phone or Zoom, during which baseline PA and technology attitude measures were taken, and verbal instructions were shared on using the smart speaker, PA app, and Wi-Fi hotspot. Participants were randomly assigned to a research team member who assisted with equipment setup and conducted all interviews and weekly check-ins throughout the intervention. Functionally, this meant that each researcher worked with the same 5 to 6 individuals throughout the study. At baseline (T0), participants took part in a brief individual motivational coaching session to determine their PA goals.

The intervention then took place in 2 phases. During phase 1, which lasted 6 weeks, participants were encouraged to use the smart speakers for their purposes (eg, answering questions, playing media, setting timers or alarms), as well as use the PA application for a minimum of 3 sessions per week. Participants were contacted weekly by phone by a trained member of the research team during this phase to troubleshoot, assess PA goal achievement, and set new PA goals for the coming week. Phase 2 lasted 4 weeks, during which participants continued natural use of the smart speaker and the PA application on their own (ie, without weekly contact).

Assessments of the participants’ perceptions of technology acceptability, prior technology experience, and attitudes toward technology were administered by the researchers at 3 points in the study: T0, 6 weeks and end of phase 1 (T1), and 10 weeks and end of phase 2 (T2). Semistructured interviews provided perceptions and use of the smart speakers and the PA application at T1 and T2. Device logs were maintained and reviewed for the entire study period. Participants were compensated after each interview (T0, T1, and T2) and received a completion bonus for completing all visits.

### Ethical Considerations

The study protocol was approved by the institutional review board of the University of Illinois Chicago (UIC Protocol 2019-1013), which reviews and approves human subjects research in accordance with the ethical principles outlined in the Belmont Report and the DHHS regulations 45 CFR Part 46. All procedures were carried out as specified in the study protocol. All participants provided oral acknowledgment of informed consent, as a written acknowledgment requirement was waived because of the conditions of the pandemic. All data were deidentified before analysis. Participants were compensated up to US $100 for time spent and were able to keep their smart speaker device on the study’s conclusion. The reporting of the qualitative findings follows the guidelines outlined in the Consolidated Criteria for Reporting Qualitative Research [56] for reporting health-related studies, as appropriate.

### Data Sources

#### Measures of Technology Acceptability and Feasibility

Researchers administered a questionnaire with quantitative measures of familiarity with technology, UTAUT, perceived sociability, and social presence during the participant interviews at T0, T1, and T2. The UTAUT subscales consisted of 30 items related to performance expectancy, effort expectancy, attitudes toward technology, social influence, facilitating conditions, smart speaker self-efficacy, anxiety, and behavioral intention to use smart speakers, measured on a 7-point scale and adapted to the context of smart speakers [31,39]. Four items were used to measure the perceived sociability of smart speakers, and 5 items assessed the social presence of smart speakers [57]. Items for each measure are listed in Multimedia Appendix 2. To indicate technological competence in everyday living, a familiarity with technology measure was adapted from the Everyday Technology Use Questionnaire [58] regarding the frequency of use of 11 everyday technologies, such as searching the internet for information, dealing with recorded telephone menus, or sending and receiving emails [59].

#### Log Data

Device log data were collected from the Google accounts attached to the devices; however, data from 2 (11%) of 18 participants was missing because of technical errors. The remaining (16/18, 89%) data logs were analyzed for frequency of engagement with the PA app by calculating the number of times “Healthy Moves” was activated by the participant. To avoid fatigue, the PA program application asked participants if they needed to take a break or stop the exercise activity. Consequently, because participants were able to voluntarily truncate the application, we used its activation as a measure of engagement.

#### Interviews and Field Notes

A total of 36 interviews were transcribed for analysis, with an average duration of 18:05 minutes for interviews at T1 and 17:06 minutes for interviews at T2. The interview guide is presented as Multimedia Appendix 3. In addition, 90 sets of field observation notes were recorded after the weekly motivational sessions during phase 1 and examined.

### Sample

Because recruitment took place during pandemic lockdowns**,** participants were recruited using social media postings, recruitment emails posted on listservs, and through the use of 2 research recruitment matchmaking portals. Inclusion criteria were those aged ≥65 years and who spoke English, as the smart speaker application was programmed in English. Participants were excluded if they participated in ≥150 minutes of PA per week or scored <7 correct responses on the Short Portable Mental Status Questionnaire [60,61].

A total of 24 participants were enrolled at T0. The size of the sample was largely determined by the availability of respondents and the adequacy of resources to complete the study. Because of interest in maintaining a PA regime and health complications, 4 participants did not complete the T1 interview. An additional 2 participants did not complete the T2 interview because of withdrawal from the study, so the final sample consisted of 18 individuals. The final study completion sample had an average age of 75.9 (SD 7.3) years; 15 (83%) of 18 participants were female, and 17 (94%) of 18 were White. Familiarity with technology was high among these participants, with 14 (78%) of 18 using a range of everyday technologies, such as searching the internet, using email, or sending text messages on a mobile device, on average, more than once per month. Table 1 summarizes the characteristics of both the study completion sample (n=18) and the recruited sample (n=24).

**Table 1:** Participant characteristics.

Characteristics			Study completion (n=18)		Entire recruited sample (n=24)
Age (y), mean (SD)			75.9 (7.3)		77.0 (8.0)
**Gender, n (%)**					
	Women	15 (83)		18 (75)	
	Men	3 (17)		6 (25)	
**Racial identity, n (%)**					
	White	17 (94)		22 (92)	
	Black	1 (6)		2 (8)	
**Education, n (%)**					
	High school education or less	3 (17)		3 (13)	
	College degree or less	9 (50)		13 (54)	
	Postcollege education	6 (33)		8 (33)	
**Income (US $), n (%)**					
	≤50,000	8 (44)		11 (46)	
	50,000-100,000	6 (33)		7 (29)	
	≥100,000	4 (23)		4 (17)	
	Not reported	0		2 (8)	
**Familiarity with technology^a^, n (%)**					
	Low (0-33)	4 (22)		8 (33)	
	High (34-55)	14 (78)		16 (67)	

^a^Adapted from Everyday Technology Use Questionnaire [58,59].

### Analysis

Data were analyzed using concurrent mixed analysis [62], with log data, interview, and field note data examined for complementarity and completeness [63] to broaden and enrich the understanding of the quantitative measures of device feasibility and acceptability. Because of this choice of method, only data collected from the study completion sample were used in this analysis. SPSS (version 29) was used for repeated measures ANOVA, and MaxQDA 2022 (version 22.08.0; VERBI GmbH) was used for first-level descriptive and second-level thematic qualitative text analysis of interview and field observation data.

The coding schema for qualitative analysis of the interview and field note date was developed using a hybrid approach, with a priori codes from the UTAUT constructs comprising the initial coding structure and additional codes developed in vivo. First, a descriptive analysis of the texts was performed by a team of 2 researchers, coding approximately 9 transcripts each. Salient words and phrases were highlighted, and special attention was paid to adjectives and adverbs that added emphasis or provided a judgment of value. Next, keywords and phrases were grouped under thematic umbrellas that either aligned with the preestablished categories derived from UTAUT codes or newly identified categories that emerged during the first descriptive coding phase. The collected measures were then grouped and analyzed according to the criteria for assessing BIT [7,44] to provide an overall assessment of acceptability, adoption, appropriateness, and feasibility. Acceptability was assessed using the UTAUT subscale of performance expectancy, along with measures of perceived sociability, pleasantness, and social presence. Adoption was measured using device log data on intervention app use and examination of the subscale on the behavioral intention to use the smart speaker. Appropriateness was evaluated using measures of attitudes toward using smart speakers and smart speaker self-efficacy. Feasibility was appraised using the UTAUT subscale of effort expectancy.

## Results

As the data analyses were conducted concurrently using mixed methods, the results are also presented concurrently. In this format, log data, interview data, and field observation data offer complementarity and completeness to the quantitative measures of acceptability and feasibility.

### Acceptability

Adults in this study perceived the smart speaker as acceptable, with measures of performance expectancy (ie, perceived usefulness) to be high during the study period (T0: mean 5.81, SE 0.16; T1: mean 5.04, SE 0.31; T2: mean 5.02, SE 0.38). However, perceived usefulness decreased over time (*F*_2,34_=3.66; *P*=.04; partial eta squared=0.18), perhaps reflecting the practicality of actual use once participants integrated the smart speakers into their everyday routines.

Participants expanded on these perceptions in the interviews, noting that specific features of the smart speaker encouraged these perceptions of utility and spurred them to engage in more frequent and routine use of the PA program. One factor they noted was that because the smart speaker was located at home, barriers to PA engagement were reduced:

Convenience. I didn’t have to go out of the home for the exercise.

Participant 8

Its accessibility and yeah, I guess that would be it. Its accessibility. It’s right there and it’s easy to consult and makes it easier to engage in the [PA] program.

Participant 19

The smart speaker also afforded participants time flexibility, enabling them to engage in the intervention at times that were convenient or available for them instead of a set or designated time. This time flexibility enabled them to adhere to the protocol with greater success:

I tried unsuccessfully to pick a time of day that worked the best and stick with it... In a way, it was an advantage because I could fit it in whenever it occurred to me or that I was reminded in some way.

Participant 5

The visual and physical presence of the smart speaker device also encouraged intervention adherence for participants, as it “reminded” participants to engage in the intervention activities, which also improved adherence:

I like the fact that it reminds me, just seeing it reminds me to do it, and I don’t know what I would use in its stead.

Participant 5

Several participants noted that the VUI interface of the smart speaker added to the device’s convenience by making it expedient to use:

I don’t have to type anything in, I can just talk to it and it gives me the quick answer that I’m looking for… I think it’s faster than the computer. Because it’s quicker to talk than for me to type it.

Participant 9

Participants perceived the smart as being pleasant to interact with throughout the study period (T0: mean 5.89, SE 0.16; T1: mean 5.27, SE 0.31; T2: mean, 5.03, SE 0.38), but this perception decreased over time (*F*_2,34_=3.63; *P*=.04; partial eta squared=0.18), perhaps because the novelty of interaction diminished over time. As one participant noted:

So I think it’s a novel way of getting one to focus on an exercise program like this, and to be able to look, shall we say, look forward to doing it, more than if it were simply something you were reading from a pamphlet.

Participant 13

They also perceived low social presence of the device (T0: mean 3.82, SE 0.27; T1: mean 3.52, SE 0.34; T2: mean 3.31, SE 0.35), and the low social presence did not change over time (*F*_2,34_=1.27; *P*=.29; partial eta squared=0.07).

### Adoption

Adoption of the smart speakers, or the intention to use them, was evidenced through actual use of the devices and indication by participants of a willingness to use its functionality for other purposes in addition to the PA intervention. Examination of the device log data revealed that while heterogeneity existed in the PA program engagement, the use of the devices decreased over the study period. For the first 6 weeks (phase 1), participants engaged with the intervention app <2 times per week (mean 10.19, SD 13.26; range 0-42). Activity was higher during this initial period, perhaps because of the accountability provided by the weekly check-in calls from the research team. Engagement with the intervention app dropped off significantly during the second phase of the study, with participants engaging with the intervention app approximately about once per week (mean 4.94, SD 7.85, range 0-29; *F*_1,15_=9.49; *P*=.008; partial eta squared=0.39). However, the patterns of use were heterogeneous, with some participants engaging with the device frequently and others only minimally.

About half of the participants engaged with the PA intervention less than one time per week during the intervention and follow-up phases (n=9), but other participants (n=4) were quite active, engaging with the intervention >4 times per week during phase 1, and continued engagement with intervention >2 times per week during the phase 2. The most frequent user engaged with the PA intervention every day during phase 1 and even more than once daily during phase 2. Examination of the interview data reinforced these findings and demonstrated how these 2 clusters differed. It also reinforced the understanding that as expectations of the device met user experiences, regular use of the devices was reinforced. As one participant noted:

Well, at first I just used it for the exercise program, and then I was a little bit more daring and listened to some music, and then jokes, and the weather, and timer and things like that. I became more comfortable using it more often.

Participant 1

Conversely, when participants did not use the device with any regularity for either exercise or everyday activities, they were less likely to indicate that they would continue using it at all. The lack of engagement with the smart speaker appeared to lead to a lack of adherence to the intervention protocol:

[B]ut I’m a little distressed with myself for not even thinking of it this week, yeah. Well it didn’t seem to be too helpful to me when I was using it, I guess that’s why I forgot about this week.

Participant 2

Participants demonstrated a strong willingness to continue using the device during and after the study period (T0: mean 6.35, SE 0.11; T1: mean 5.98, SE 0.27; T2: mean 5.90, SE 0.32), and this behavioral intention to use the device did not change over time (*F*_2,34_=1.48; *P*=.24; partial eta squared=0.08). We note that participants who made a concentrated effort to incorporate the device into their daily routine during the study, for both exercise and other activities, demonstrated greater intention to continue using the device, even beyond the scope of the study.

### Appropriateness

Appropriateness, or the perception that the technology is compatible with the context of its use, was demonstrated consistently during the study period, though these perceptions were not uniform among participants. Participants held strongly positive attitudes toward smart speakers (T0: mean 6.17, SE 0.09; T1: mean 6.07, SE 0.16; T2: mean 6.00, SE 0.18), and these perceptions were sustained throughout the study (*F*_2,34_=.82; *P*=.45; partial eta squared=0.05). Comments about specific qualities of the smart speakers that enhanced the delivery of the PA intervention elaborated on these positive perceptions. For example, one prominent feature that was frequently mentioned by participants was the VUI. Participants found the auditory instructions provided by the smart speaker to be an appropriate mechanism to deliver the PA intervention, likening it to an individual coach or mentor:

Well, I do like the fact that even though this is not a real person, there is a voice telling you what to do. So you’re not in a gym filled with people or you’re not in a class. You don’t have to go anywhere, but someone is standing there telling you what to do or sitting there telling you what to do. And so that is a, I think for persons who say live by themself or something, it is like another voice that, I guess it’s watching a TV too, but it’s a voice that coaches you on.

Participant 24

Other participants liked the ability just to listen and follow instructions. The simplicity of following commands enabled them to carry out the activities easily and with minimal cognitive effort:

I don’t have to think about it because I just follow whatever the directions are. When we’re doing the other exercise, I have to use the paper and I have to look at the paper. In here, you just follow the directions and you’re all set.

Participant 7

However, the VUI may also have presented some hindrances for delivering the PA intervention. Prior work has identified that older adults may experience difficulty with constructing a structured sentence command in smart speaker use [29]. Participants in this study described how they had to “learn to talk to the device” by rephrasing questions and commands to obtain a desired response:

But every now and then on the [PA application], I think you’re supposed to answer a certain way. If he [the smart speaker voice] says, “Are you ready to go to the next exercise?” Then you go “Uh-huh.” And he’s like, “Did you hear me?” I think maybe you should know the respect that he needs because sometimes I think he doesn’t understand me and I forget to speak clearly.

Participant 11

Participants consistently perceived that they could effectively use the smart speakers, assessing their self-efficacy in using the smart speakers quite positively (T0: mean 6.03, SE 0.12; T1: mean 5.79, SE 0.26; T2: mean 5.58, SE 0.25). However, this self-efficacy assessment dropped during the study period (*F*_2,34_=1.81; *P*=.18; partial eta squared=0.10) as participants integrated the devices into daily routines. Self-efficacy was also demonstrated by participants’ ability to engage in intuitive workarounds for issues of communication with the smart speakers. These workarounds consisted of repeating or rephrasing commands, adjusting the speaking volume, or articulating the command more clearly:

...I don’t know whether it was the way I asked, ya know, I asked the question and the device said, “I didn’t understand the question.” So I said it a different way and then it was able to answer.

Participant 7

Well, once in a while, I think maybe I’m not close enough to it and they will say, “I can’t understand you.” And I don’t know whose fault that is, if it’s a Mini Google device or maybe I’m not directly in front of it. But then I try again and I’m able to interact with it.

Participant 12

However, challenges with the ability to engage with the intervention on the smart speaker may have contributed to participants’ feelings of being less proficient in using the smart speaker as the study progressed. Miscommunication issues with the device appear to be the primary hindrance and sometimes result in the participant ceasing interaction with the smart speaker or taking time away from the device before attempting interaction again. This effectively prevented the participant from carrying out the intervention at the desired or appropriate time:

Sometimes the Google device misses the mark as far as giving me exactly what I’m looking for. So I either have to rephrase what I’m asking or give up.

Participant 13

Other participants, especially those who used the device irregularly or only for the PA application, were often discouraged when faced with these difficulties. Some participants did not seem comfortable adjusting their language or speaking style to accommodate the device and would disengage from the conversation and walk away:

[B]ut when I tried to get onto the [PA application], I tried it twice and then both times it said it wasn’t responding, but I didn’t get frustrated. I just thought, “I’m not going to do exercises today.”

Participant 1

### Feasibility

Feasibility, or the perception that smart speakers are easy to use, remained high during the study period***.*** Participants reported that they found the smart speakers easy to use (T0: mean 5.93, SE 0.53; T1: mean 5.56, SE 1.16; T2: mean 5.55, SE 1.24), and this ease of use was sustained throughout the study (*F*_2,34_=1.49; *P*=.24; partial eta squared=0.08). As one user summarized in the interviews:

I found it very easy to use. Very, very helpful. It got me to exercise three times a week, more than I could have without it.

Participant 1

From the perspective of the research team, however, feasibility was not as clear cut. Examination of field note observations revealed that during the weekly contacts in phase 1, members of the research team often assisted participants in effectively using their smart speakers. Though participants reported that their devices were easy to use, researchers noted that they had made suggestions that devices be moved within the participant’s residence to improve the Wi-Fi signal (n=5) and that participants might rephrase commands to the device to gain an appropriate response (n=5), or worked with participants to adjust volume settings (n=2). In addition, researchers suggested and encouraged participants to use the device in alternate ways in addition to the PA program app (n=4), such as seeking information about the weather or setting timers and alarms. The frequency and nature of these observations suggest that providing support for using the devices was a necessary element in the research protocol and required resources from an administrative perspective, including training members of the research team in basic device functionality as well as potential troubleshooting strategies.

Wi-Fi connectivity issues were a major determinant of the problems that participants experienced. When smart speakers have difficulty maintaining a continuous internet connection, especially when deployed over Wi-Fi hotspots, they may encounter limits on bandwidth, which can impair their operation [64]. The use of Wi-Fi hotspots was a source of some of the connectivity issues experienced by participants and caused frustration in the form of longer-than-anticipated response times or interruptions in the performance of the device. These signals of connectivity issues required patience when experienced:

I would be patient. Sometimes there’s a long pause before it responds. So patience is sometimes necessary, or just repeat my request. And if I get a sense that it’s not being digested by the device, I rephrase it.

Participant 13

The placement of the smart speaker within the participant’s residence was also a critical factor in ensuring successful interactions with the device. Prior research has identified that older adults strategically position smart speakers to shape and organize daily routines [65]. Participants frequently placed devices in the area of their residence, which they spent the most time in to optimize accessibility and convenience. Often, this meant that the smart speaker was placed in a living or family room, but the kitchen was also a popular option. However, these spaces were not always optimal for participating in the PA program intervention. As one participant noted:

Now, I’m sitting in a computer room a study. And so, a lot of times I do it [the PA program application] in here. In fact, I think I’ve been doing it in here for the last week or two, but sometimes I put it [the smart speaker] in the area where we eat, the kitchen eating area, so that if I’m working in the kitchen and I’d like to use it for a timer. So, then I, because it’s there, I ended up doing the exercises in the kitchen.

Participant 16

Privacy considerations also play a role in device location and, again, may run counter to optimal placement for engagement with the intervention activity or a strong Wi-Fi signal. As one participant described:

My husband and I worry about the privacy issues with the Google Home Mini, and several times when I had it plugged in to prepare in order to do exercises, I would mention, “Oh, I have to Google that.” And the Google Mini would come on. And that was very disconcerting because I was in another room and it was listening.

Participant 21

Ultimately, participants seemed to maximize the opportunity to use the smart speakers and tried to place the device in ways that would facilitate their engagement with the intervention:

I kept it right here in the living room where it was visible to me every day. If I had tucked it away somewhere, that would have been even more problematic on my part because it’s easy to forget.

Participant 5

### Fidelity

Fidelity, or evidence that the technology can deliver the intervention as intended, is a criterion that is perhaps best viewed from the perspective of the research team, but participants noted how the smart speaker enabled them to execute the intervention with greater precision. Participants liked the program’s routine and noted that it provided structure, including PA, in their daily routines.

I like the fact that it times you, in other words, I don’t have to keep track of the time with the clock or anything like that. I like, it just gives a little more structure so that I can rely less upon my own motivation. I would say it serves as a motivator too.

Participant 9

In addition, some participants noted that the smart speaker introduced accountability into their engagement with the intervention, alluding to its potential to be used as a mechanism to report on activities associated with the study:

And I realized that I’m on my own time. I can do this or I don’t have to do it, but having the device makes me feel responsible that it’s kind of like big brother is watching me, and if I don’t use it, it’s going to know.

Participant 18

Though the PA application had been tested extensively before deployment in the study, issues with its performance and interoperation with the device were encountered during both phases of the study. Participants reported that they experienced technical difficulties with the PA application, including the application not responding to them in a timely manner, aborting the intervention without warning, or repeating exercises that they had already completed. Minor modifications to the application by the development team during the study resulted in some improvements in performance, but most incidents were attributable to Wi-Fi connectivity issues, which could cause the PA program application to cease functioning and impair the fidelity of the intervention.

Participants found incidents very frustrating and for some, induced nonuse of the intervention application. As one user stated:

I tried to do it with the (PA application), but it doesn’t work right for me, so I just don’t use it then. So, I do a little exercise, but I just don’t use the Google, I just do them on my own.

Participant 20

As this participant suggests, when encountering technical issues with the PA application, participants often maintained the exercise regimen of the study without the assistance of the smart speaker. Participants had received extensive support materials in their orientation packet, including illustrated descriptions of the activities that were invoked by the PA application. Because of these materials, several participants felt that they could complete the exercises without the guidance of the smart speakers; however, some used the functionality of the device, such as a timer, to assist in carrying out their activities:

[Participant] told me when she gets frustrated with that she will just use the Google Home [smart speaker] to set a timer and go through the exercises on her own.

Field notes, Participant 16

Participants also appeared to be able to distinguish between the PA application and the hardware of the smart speaker when they reported the challenges they encountered. This was made clear through their use of gendered pronouns when speaking about the various components, identifying the male voice of the PA application and the female voice of the smart speaker interface, as well as specific references to the PA intervention as an “app.”

Well, there were problems with the software where it would abort and I would be shifted over to the regular Google application as opposed to the [name of PA program app].

Participant 13

Taken together, these last 2 points suggest that when participants’ challenges with the PA application were technical, they recognized it was a limitation of the intervention delivery mechanism and not the smart speaker or the intervention. Instead, they sometimes resorted to workarounds, such as relying on their memory to complete the exercise or using the support materials as a prompt. This underscores the importance of providing support materials to participants, not only for the delivery mechanism but also for the intervention itself.

## Discussion

### Principal Findings

The goal of this paper was to evaluate the use of smart speakers to deliver an in-home PA intervention among a sample of older adults using previously established criteria for the evaluation of BITs. Our focus for this analysis was on the smart speaker as a delivery mechanism and not the PA intervention itself, as these are distinct components of the intervention implementation and should be evaluated separately. The smart speakers in this study were found to be highly rated for a PA program by participants regarding acceptability, appropriateness, and feasibility, criteria essential to quality BITs [7,44]. Functional and technical factors related to the operation of the smart speakers, such as ensuring consistent Wi-Fi connectivity and ensuring participants used appropriate phrasing when interacting with the devices, created a responsibility for the research team to provide basic technical support and troubleshooting resources. In addition, these same factors possess the potential to impair the fidelity of the intervention. In short, while smart speakers provide a novel and acceptable technology for intervention research, their feasibility in a research context comes with limitations.

Smart speakers afforded participants in this study convenience and flexibility for engaging with the intervention activities and served as a visual reminder to reinforce completion of the study protocol, which improved adherence to the intervention. The VUI was well-received by participants, who noted its ease of use and appropriateness for coaching participants through a PA program. The VUI also introduced challenges for the participants, as it required them to learn how to appropriately phrase commands and adjust their speaking volume to communicate with the device. This represents an intriguing intersection of possibility and limitation. On the one hand, the benefits of the device, as articulated by most respondents, are based on its ease of use and convenience, which are associated with being a hands-free interface that is capable of responding quickly and specifically. This presents a range of possibilities for intervention-based research across health, education, and other applications for older adults [66,67].

By contrast, at the current stage of development, the smart speakers used for this study are not capable of accommodating a human user’s natural diction and phrasing beyond stating “I’m sorry” and requesting the user rephrase their question or direction until an acceptable rudimentary keyword or phrase is recognized. Therefore, successful accommodation to miscommunication hinges on the ability of the participant to mold their habits and language to patterns recognizable by the device. When participants were flexible about adjusting their phrasing and behaviors to mitigate technical glitches, they were also more likely to view the device favorably and use it regularly. When considering acceptability, this is particularly meaningful because the limitations of the technology and the adaptiveness of the participant base must be evaluated in tandem. Future development in artificial intelligence–supported health interventions could leverage the advancement in large language models to provide ubiquitous and fluent user experience [68].

Participants indicated their intention to use a smart speaker through positive attitudes toward its functionality, but there was a high degree of heterogeneity in their adoption. Some participants embraced the use of the devices, whereas others were frustrated and abandoned their efforts easily. Issues related to Wi-Fi connectivity were particularly challenging and interfered with the ability of the device to function appropriately. The feasibility of using smart speakers in an intervention, while positive from the participants’ perspective, was more challenging from the vantage point of the research team. To provide technical and operational support for successful device operation, members of the research team were required to be familiar with device functionality and basic troubleshooting strategies. In addition, because participants had discretion over device placement within their residence, it was more challenging to ensure that the device would be optimized for both execution of the intervention and Wi-Fi connectivity. These connectivity issues impacted intervention fidelity and underscored the need for robust support.

Overall, technical issues, such as glitches with the PA application, device-level technical problems (volume, articulation, etc), and broader critical infrastructure issues, such as a weak Wi-Fi signal, will stymie engagement with intervention activity and broader intentions to use the smart speakers. However, when participants have outlets to seek technical assistance and support when issues arise, such issues can be effectively mitigated. In other words, older adults are not at all resistant to engaging with smart speakers; however, a robust technical and informational support system should be in place.

The privacy considerations for smart speakers are not inconsequential. To be activated, smart speakers typically require an account to be established with the device maker, which is then associated with the smart speaker. Often, these accounts require additional information to be gathered from the user, such as credit card details, causing concern about personal information collection [69]. Establishing a linkage between the account and the smart speaker enables personalization of the activities that can be performed, such as reminders; however, it also associates this same information with voice recordings and device interactions [13]. Location-based data, such as time zone and zip code, allow device makers to transmit relevant information, such as weather and traffic news, but these data also become associated with accounts. All of these additional data points may increase participant privacy vulnerability.

One strategy to enhance participant privacy is to use pseudonym accounts to set up the smart speaker and collect data, as was done in this study. This approach provides some ability to shield participants’ identity, but it also reduces the functionality of smart speakers considerably, as devices are unable to be personalized for calendaring and reminding functions. This reduction in utility can be frustrating to study participants, who anticipate a level of functionality frequently advertised by device makers. However, ultimately, privacy concerns are associated with smart speaker use [70], and those who use them may be qualitatively different than the general population [71], thereby presenting a form of sample selection bias for research using the devices.

### Limitations

Data analyzed in this study were collected from a relatively small, not randomly controlled sample, so the conclusions reached may not be generalizable to a wider older adult population. This study was conducted during the pandemic, which required recruitment to be carried out via the web, and interviews were conducted via telephone or Zoom. The implications of this context are 2-fold. First, this sample may have been more accepting of technology and may have had higher educational attainment than if they had been enrolled in a clinical or face-to-face setting. This might suggest that the issues highlighted with this sample may be even more pronounced with populations with lower technological proficiency or education levels and may require researchers to provide additional support resources when carrying out studies of this nature. Second, the context of the pandemic may have prompted the participants to have greater acceptance of smart speaker technology to engage with PA, as in-person activities were significantly reduced during that time. These factors also limit the generalizability of these findings as they may have introduced a positive bias to perceptions of acceptability and feasibility in this sample. To gain a greater and more nuanced understanding of the acceptability and feasibility of using smart speakers in a research context, additional studies with representative samples in a nonpandemic context are required.

Challenges encountered by participants that were related to the wireless connectivity of the smart speakers may also have influenced perceptions of the smart speaker and the intervention application. Further exploratory work is needed to distinguish how perceptions of applications can be distinguished from their related delivery mechanisms for evaluation of usability, feasibility, and efficacy. In addition, data collection was constrained because of the pandemic, which limited the ability to observe participants engaging with the smart speakers. Further work is needed to expand data collection efforts beyond self-reports, which would offer additional perspectives on the acceptability of smart speakers in intervention research and provide greater detail on evaluation criteria, such as fidelity.

Finally, interviews at T1 and T2 were only conducted with participants still enrolled in the study at those time points. While data were collected from individuals who did not adhere to the intervention, it did not include input from those individuals who did not complete the study. This factor limits the interpretability of these findings, as input from noncompleters may have included perceptions related to a lack of feasibility or acceptability of smart speakers for use in a PA program.

### Conclusions

In conclusion, findings from this study found smart speakers to be acceptable and appropriate for PA intervention research involving older adults, with participants indicating a willingness to adopt these delivery mechanisms for the delivery of the intervention program as well as for their everyday use. However, the feasibility of these devices for use in research contexts was mixed as they require specific and specialized attention to technical support and troubleshooting when used with older adults. Finally, applications developed to run on smart speakers must be developed to minimize disruption, whether because of flaws in design or through careful planning related to the overall Wi-Fi infrastructure, as weakness in this capacity may impair the ability of smart speakers to deliver interventions with high fidelity.

This study contributes to intervention research in that it evaluates the acceptability and feasibility of smart speakers as a behavioral intervention delivery infrastructure or the mechanisms through which an intervention is delivered, separately and distinctively from the technology that comprises an intervention, which here was a PA program application designed to enhance the physical well-being of sedentary older adults. Conducting separate evaluations of these intervention delivery elements is necessary to ensure a thorough assessment of intervention outcomes. Results from this study highlighted that older adults perceive smart speakers to be useful and easy to use. Future studies might explore the suitability of smart speakers as a delivery infrastructure for aspects of behavioral interventions requiring smart speaker functionalities, such as the setting or reminders or the streaming of media content. Research on the use of smart speakers in other specialized populations, such as those with visual impairment or limited mobility, may also prove fruitful.

In addition, these findings offer important insight for research practitioners. At the very basic level, it cautions against oversimplifying the implications of using complex delivery infrastructures, especially with a population such as older adults that might lag the general population in the adoption and use of emerging technologies. On one level, such oversimplification may overlook important aspects of technological delivery mechanisms, such as the provision of technical support and troubleshooting, which can often tap into limited research resources. On a more granular level, the same functional and technological issues that prompt the need for support resources, such as ensuring continuous Wi-Fi connectivity, can ultimately negatively impact intervention fidelity and compromise the integrity of the research process. Taken together, smart speakers offer a novel delivery infrastructure for behavioral intervention research but also require careful planning.

## Appendix

Multimedia Appendix 1 Activities of the physical activity program.

Multimedia Appendix 2 Measures of technology acceptability and feasibility.

Multimedia Appendix 3 Interview guide.

## Abbreviations

BIT: behavioral intervention technology
PA: physical activity
UTAUT: Unified Theory of the Acceptance and Use of Technology
VUI: voice user interface

## Data Availability

The data sets generated during and analyzed during this study are available from the corresponding author on reasonable request.
