# Correction: Assessing the Feasibility and Acceptability of Smart Speakers in Behavioral Intervention Research With Older Adults: Mixed Methods Study

**DOI:** 10.2196/66813

**Published:** 2024-11-15

**Authors:** Kelly Quinn, Sarah Leiser Ransom, Carrie O'Connell, Naoko Muramatsu, David X Marquez, Jessie Chin

**Affiliations:** 1 Department of Communication University of Illinois Chicago Chicago, IL United States; 2 Division of Community Health Sciences School of Public Health University of Illinois Chicago Chicago, IL United States; 3 Department of Kinesiology and Nutrition University of Illinois Chicago Chicago, IL United States; 4 School of Information Sciences University of Illinois Urbana-Champaign Urbana, IL United States

In “Assessing the Feasibility and Acceptability of Smart Speakers in Behavioral Intervention Research With Older Adults: Mixed Methods Study” (J Med Internet Res 2024;26:e54800) the authors would like to make an addendum to the Acknowledgements:

The corrected text should read:

This work was supported by the the National Center for Advancing Translational Sciences, National Institutes of Health [UL1TR002003] and the National Institute on Aging of the National Institutes of Health [R24AG064191, P30AG022849]. The content is solely the responsibility of the authors and does not necessarily represent the official views of the National Institutes of Health. The funders had no role in the design and conduct of the study; collection, management, analysis, and interpretation of the data; preparation, review, or approval of the manuscript; or decision to submit the manuscript for publication. The authors would like to thank Smit Desai for his contribution to an earlier version of the manuscript. We also would like to thank the following individuals for their assistance with this project: Smit Desai, Jamie Gradishar, Daniel Hlavacek, Faraz Hussain, Caniece Leggett, Fernando Mendez, and Melissa Pierce.

The correction will appear in the online version of the paper on the JMIR Publications website on November 15, 2024, together with the publication of this correction notice. Because this was made after submission to PubMed, PubMed Central, and other full-text repositories, the corrected article has also been resubmitted to those repositories.

