# Supplementary material for: Assessing the Feasibility and Acceptability of Smart Speakers in Behavioral Intervention Research With Older Adults: Mixed Methods Study
Source: J Med Internet Res. 2024 Aug 30;26:e54800. doi: 10.2196/54800 (PMC11399739; doi:10.2196/54800)
Supplement: Multimedia Appendix 1 [file jmir_v26i1e54800_app1.docx]

**Multimedia Appendix 1: Activities of the Physical Activity Program**

Participants were introduced to the PA program application in an orientation session with one of the researchers. The following activities were included in the applications:

Seated exercises:

- Arm curls with a one-pound weight - 15 to 20 repetitions on each arm
- Ankle Point & Flex - 30 second hold on each foot
- Seated Step-In-Place - one minute
- Chair Stand - 10 repetitions

Standing exercises:

- Standing Step-In-Place - 15 to 30 seconds
