# Supplementary material for: Assessing the Feasibility and Acceptability of Smart Speakers in Behavioral Intervention Research With Older Adults: Mixed Methods Study
Source: J Med Internet Res. 2024 Aug 30;26:e54800. doi: 10.2196/54800 (PMC11399739; doi:10.2196/54800)
Supplement: Multimedia Appendix 2 [file jmir_v26i1e54800_app2.docx]

**Multimedia Appendix 2: Measures of Technology Acceptability and Feasibility**

**Familiarity with Technology**

Responses are based on a five point scale, with 1=Never and 5=More than once a day.

 Please indicate how often you:

1. Search for information on the internet
2. Use the TV remote control
3. Withdraw money from an automatic teller machine
4. Deal with recorded telephone menus
5. Record a TV program using a device
6. Send and receive emails
7. Use a mobile phone
8. Access the internet on your mobile phone
9. Operate a telephone answering service such as an answering machine or voicemail
10. Use a microwave oven
11. Send and receive text messages on your mobile phone

**Unified Theory of Acceptance and Use of Technology (UTAUT)**

For the following subscales, responses are based on a 7-point Agree-Disagree Likert scale.

1. **Performance expectancy**
   1. I would find a [conversational agent] device useful.
   2. Using a [conversational agent] device enables me to accomplish tasks more quickly.
   3. Using a [conversational agent] device will make me more productive.
2. **Effort expectancy**
   1. My interaction with a [conversational agent] device would be clear and understandable.
   2. It would be easy for me to become skillful at using a [conversational agent] device.
   3. I would find a [conversational agent] device easy to use.
   4. Learning to operate a [conversational agent] device is easy for me.
3. **Behavioral intention to use**
   1. I intend to use a [conversational agent] device in the next <n> months.
   2. I predict I will use a [conversational agent] device in the next <n> months.
   3. I plan to use a [conversational agent] device in the next <n> months.
4. **Attitude toward using technology**
   1. Using a [conversational agent] device is a bad/good idea.
   2. A [conversational agent] device makes things more interesting.
   3. Using a [conversational agent] device is fun.
   4. I like using a [conversational agent] device.
5. **Smart speaker self-efficacy**
   1. I could complete a job or task using a [conversational agent] device...
   2. If there was no one around to tell me what to do as I go.
   3. If I could call someone for help if I got stuck.
   4. If I had a lot of time to complete the task for which I was using it.
   5. If I had just the built-in help facility for assistance.

**Perceived Sociability**

Responses are based on a 7-point Agree-Disagree Likert scale.

1. I consider [conversational agent] to be a pleasant conversational partner.
2. I find [conversational agent] pleasant to interact with.
3. I feel [conversational agent] understands me.
4. I think [conversational agent] is nice.

**Social Presence**

Responses are based on a 7-point Agree-Disagree Likert scale.

1. When interacting with [conversational agent] I felt like I was talking to a real person.
2. It sometimes felt as if [conversational agent] was really listening to me.
3. I can imagine [conversational agent] to be a living person.
4. I often think [conversational agent] is not a real person.
5. Sometimes [conversational agent] seems to have real feelings.
