# Supplementary material for: Assessing the Feasibility and Acceptability of Smart Speakers in Behavioral Intervention Research With Older Adults: Mixed Methods Study
Source: J Med Internet Res. 2024 Aug 30;26:e54800. doi: 10.2196/54800 (PMC11399739; doi:10.2196/54800)
Supplement: Multimedia Appendix 3 [file jmir_v26i1e54800_app3.docx]

**Multimedia Appendix 3: Interview Guide**

Tell me what it’s like to have the smart speaker at your home.

Think back to the last time you used the smart speaker; can you tell me about that? What do you use it for?

In your daily life, when do you use a smart speaker?

In your daily life, what activities would you like to do with a smart speaker?

Over the last week, can you think of an example of your interaction with smart speaker that you are satisfied with?

Over the last week, can you think of an example of your interaction with smart speaker that made you feel frustrated?

Think back to the last time you use the smart speaker to start the physical activity program, can you tell me about that? When? Under what condition? For how long? Any difficulties?

In your daily life, what makes you want to use the smart speaker to start the physical activity program?

Have you experienced any difficulties using the smart speaker to do the physical activity program? How do you solve it?

What do you like about using smart speaker to do the physical activity program?

What do you NOT like about the use of smart speaker to do the physical activity program?

How do you feel about your interaction with the physical activity program on the smart speaker?

How do you feel about your interaction with the smart speaker overall?

**Additional questions for T2 interviews**

Do you have any difficulty with using the smart speaker?

If your smart speaker does not work as expected, what do you usually do?

What functions do you like the best?

What functions you use most often with your smart speaker?

What makes you continue using the smart speaker?  (Or what makes you discontinue using the device?)

Can you give me some reasons that make you want to use smart speaker?

What makes you NOT want to use smart speaker?

For an ideal smart speaker, what additional functions would you like to have?
